# Oxidative Stress in SLE T Cells, Is NRF2 Really the Target to Treat?

**DOI:** 10.3389/fimmu.2021.633845

**Published:** 2021-04-23

**Authors:** Kim Ohl, Klaus Tenbrock

**Affiliations:** Department of Pediatrics, Pediatric Rheumatology, Medical Faculty, RWTH Aachen University, Aachen, Germany

**Keywords:** Keap 1, ROS, mTOR, lupus, Tregs, Foxp3

## Abstract

Oxidative stress is a major component of cellular damage in T cells from patients with systemic lupus erythematosus (SLE) resulting amongst others in the generation of pathogenic Th17 cells. The NRF2/Keap1 pathway is the most important antioxidant system protecting cells from damage due to oxidative stress. Activation of NRF2 therefore seems to represent a putative therapeutic target in SLE, which is nevertheless challenged by several findings suggesting tissue and cell specific differences in the effect of NRF2 expression. This review focusses on the current understanding of oxidative stress in SLE T cells and its pathophysiologic and therapeutic implications.

## Introduction

Oxidative stress might be a central factor in the immunopathogenesis of Systemic Lupus Erythematosus (SLE) (1). There are data supporting the hypothesis that excessive reactive oxygen species (ROS) production is, along with many other factors, one of the factors that induce SLE (2). Indeed, ROS production associates with enhanced apoptosis and might delay clearance of apoptotic cells, both of which are hallmarks of SLE (3, 4). Apoptotic cells are a source of autoantigens (e.g., ribonucleoproteins, DNA), which can be recognized by auto-reactive T cells. Furthermore, apoptotic cells release danger-associated molecular patterns (DAMPs), DAMPs are danger signals, which induce inflammatory responses. Therefore, increased auto-antigenic exposure and decreased autoantigen removal might contribute to autoantibody production and autoimmunity. Peripheral blood mononuclear cells (PBMCs), T cells and neutrophils are exposed to increased concentrations of free radicals which leads to aberrant activation of these cells. In addition to this, mitochondrial dysfunction and ROS production of PBMCs themselves contribute to SLE pathogenesis, while mechanistic reasons that cause mitochondrial ROS production have not been identified (5). It is not clear if the chronic inflammation in SLE induces oxidative stress conditions or if ROS production is vice versa a cause of lupus pathology. Nevertheless, there is mounting evidence for a defective redox clearance in SLE. Patients with highly active disease show impaired activity of oxidative stress regulating enzymes, such as superoxide dismutase (SOD) and gluthathione peroxidase (GPX) in serum and saliva while PBMCs of active SLE patients reveal higher intracellular ROS than those of healthy controls (6–9). Increased oxygen intermediates have also been identified in macrophages of lupus mice (10). Therefore, studies even suggest considering assessment of redox status as a serological marker to evaluate disease activity and nephropathy in SLE (11–13).

On the other hand, ROS production has been associated with the prevention of autoimmune diseases. A genetically mediated deficiency of ROS production can contribute to initiation of autoimmunity and can facilitate disease progressing (14). A missense mutation in the p47phox (Neutrophil Cytosolic Factor 1, *NCF1*) subunit of NADPH oxidase (NOX) predisposes to SLE and other autoimmune diseases (15, 16). Mice with a mutation of *Ncf1*, which is associated with low ROS production, develop an accelerated lupus-like disease (16). In addition to this, point mutations in *NCF2* are associated with increased SLE risk (17), since they reduce NOX activity and NCF-2 deficient or NCF-2 haplo-insufficient mice reveal accelerated lupus disease (18).

To sum up these findings, ROS play an ambivalent role in SLE, which depends on source, location, amount and most probably also time-point of their occurrence. As a consequence, balanced ROS levels are important to sustain an immune equilibrium which reduces tissue damage and prevents the development of autoimmunity (19, 20). But how is a redox balance achieved in our cells? To answer this question, we have to study NRF2, the main transcription factor of the anti-oxidative stress response.

## NRF2

Oxidative stress can activate different transcriptional regulators, most importantly NRF2. In steady state conditions, NRF2 is bound in the cytosol by Kelch ECH associating protein 1 (KEAP1), which induces ubiquitinilation and degradation of NRF2 (21). Different cellular stimuli which induce oxidative stress result in conformational changes of KEAP1, which foster the release of NRF2 from KEAP1 followed by NRF2 translocation into the cellular nucleus and activation of genes containing an antioxidant response element (ARE) in their promoter regions (22). NRF2 thereby induces expression of phase II detoxifying enzymes and antioxidant proteins, which preserve cellular homeostasis by reducing chemical or oxidative stress molecules. These proteins include enzymes mediating glutathione (GSH) synthesis, the thioredoxin enzyme system and detoxifying enzymes such as heme oxygenases, or NAD(P)H: quinone oxidoreductase 1 (NQO1). In addition to induction of antioxidant genes, NRF2 also modulates immune responses by regulating transcription of several others including anti-inflammatory and metabolic genes in immune cells (23–26). So, being a critical regulator of cellular oxidative stress responses and inflammatory reactions, it is not surprising that NRF2 is indispensable to prevent cellular damage and subsequent inflammation. NRF2 deficient mice have problems to deal with inflammatory cues and therefore show a more sever phenotype in inflammation-mediated animal models like experimental asthma (27), acute lung injury (28), sepsis (29), T cell-mediated hepatitis (30), or dextran sulfate sodium-induced colitis (31) and arthritis (32). There are also several lines of evidence that NRF2 has a central role in the pathogenesis of SLE. Interestingly, aged female NRF2 deficient mice are prone to develop an autoimmune condition that closely resemble human SLE (33). Furthermore, NRF2 deficiency increased lupus nephritis and Th17 cells in B6/lpr mice (34). NRF2 furthermore suppresses Lupus nephritis by neutralizing ROS and preventing renal damage (35). A recent study revealed that NRF2 activation promotes resolution of chronic inflammation in lupus most probably by repolarization of macrophages and reduction of the IFN signature (36). In contrast, one study revealed a prolonged lifespan, improved autoimmune nephritis, and reduced lymphadenopathy of lupus mice in the absence of Nrf2 (37). However, these effects can be explained by a Nrf2 mediated suppression of the autoimmune accelerating gene lpr, which is used in this mouse model. It is therefore not clear, how this study can be transferred to the human SLE disease. With that regard, Gautam et al. found elevated ROS in SLE specific DCs, which might be due reduced clearance of ROS related to impaired levels of NRF2 (6). In addition, the association of the NRF2 -653G/A polymorphism with lupus nephritis in pediatric-onset SLE has been described (38). This polymorphism was only associated with nephritis, while there was no significant association between NRF2 -653G/A polymorphism and susceptibility to SLE. Another study identified three SNPs and one triplet polymorphism within the promotor and upstream regions of the NRF2 gene, but no association between risk of SLE and these polymorphisms. However, authors state that their analysis is preliminary and only a small number of patients were observed (39). Therefore, until now we can only assume the association between Nrf2 polymorphism and lupus nephritis.

In conclusion, there are several lines of evidence which support the conclusion that NRF2 activation is beneficial in SLE pathogenesis. However, it is not clear whether NRF2 - by induction of the antioxidant machinery - enhances cell survival and reduces apoptosis, which contributes to the control of systemic inflammation and limitation of autoimmune responses or by direct cellular mechanism including transcriptional regulation of inflammatory responses in immune cells.

A very interesting study addressing this question was performed by Suzuki et al., who analyzed NRF2 in the scurfy mouse model. These mice lack functional regulatory T cells (T_regs_) and therefore develop mutiorgan-inflammation and autoimmunity at early ages. Interestingly, while systemic activation of NRF2 by *Keap1* knockdown ameliorated tissue inflammation, NRF2 activation *via* cell lineage-specific *Keap1* disruption (i.e., in T cells, myeloid cells, and dendritic cells) achieved only partial or no improvement in the inflammatory status of scurfy mice (40). This suggests NRF2 activation in multiple cell lineages appears to be required for sufficient anti-inflammatory effects. Our group even observed a pro-inflammatory effect of NRF2 when we specifically deleted NRF2 in T_regs_ (26). This raises the question if it is beneficial to use NRF2 activation in SLE as a therapeutic intervention. It is therefore important to first illuminate the exact role of NRF2 critically and ROS signaling in T cells in SLE.

## NRF2 and Redox Metabolism in T Cells

Redox-dependent signaling pathways are of major importance in immune cells and specifically in T cells. They control different functions including T effector cell differentiation and migration, cell cycle progression and inflammation. The role of ROS in T cell signaling has been intensively reviewed elsewhere (41). We will therefore only give a short overview about the current knowledge in this field. The production of ROS is enhanced after T cell receptor stimulation and is necessary for clonal expansion. ROS which is produced in the mitochondrial electron transport chain (ETC) is important for the activation of NFAT, a T cell-specific transcription factor that facilitates interleukin-2 (IL-2) expression and thus cell cycle progression, which results in rapid proliferation of the T cells (42). However high ROS concentrations result in GSH depletion, since the murine Glutamate-Cysteine Ligase Catalytic Subunit (GCLC) is not expressed in murine T cells. GSH depletion negatively affects NFAT activity, mTOR signaling and MYC protein expression and thus inhibits T cell proliferation (43). The antioxidant GSH tightly regulates ROS activity, which thus controls cell cycle progression of T cells as well as their metabolic properties. The latter ones influence their differentiation program and as a consequence, the manner of protective immune responses. Thus, ROS influence metabolic programming; too much can be detrimental, but if applied correctly, it is important for protection of the host (19). For metabolic activity, T_regs_ rely on oxidative phosphorylation (OXPHOS), which produces ROS and which are also regulated by GSH. GSH generated by GCLC is indispensable for proper T_reg_ function and lack of GCLC in T_regs_ results in autoimmunity (44).

Giving this critical role of ROS in T cells, it is obvious that NRF2, which regulates cellular redox homeostasis might also critically regulate T cell functions. It is known that NRF2 expression is increased in activated T cells in mice and men (26, 45, 46), however the exact role of NRF2 in T cells remains controversial. Constitutive activation of NRF2 in T cells was protective in a murine acute kidney injury model and involved higher frequencies but not total numbers of intrarenal Tregs, as well as reduced expression of inflammatory cytokines in CD4^+^ T cells (47). However constitutive ablation of NRF2 in T cells ameliorated graft-vs-host disease (GvHD) (46). Again, a study with human cells indicates that high expression of NRF2 in CD8^+^ T-cells might be protective against chronic GvHD (48). The role of oxidative stress in T_regs_ is controversely discussed. Mougiakakos et al. have been reported that T_regs_ are more resistant to oxidative stress-induced cell death than conventional T cells (49), while others have found higher toxicity of free oxygen species in T_regs_ to, which was attributed to a diminished NRF2-dependent antioxidant system (50). The weakness of the NRF2-associated antioxidant system facilitates T_reg_ apoptosis but nevertheless causes enhanced immunosuppressive capacity within the tumor microenvironment. Our own study indicates that NRF2 is a negative regulator of T_reg_ function and that Foxp3 specific activation of NRF2 results in a loss of Foxp3 expression and spontaneous accumulation of IFN-γ producing effector T cells and spontaneous inflammation (26).

Consequently, NRF2 has shown pro- and anti-inflammatory potential in T cells in different experimental inflammatory mouse models. In addition to this, there are differences between the role of NRF2 in human and murine T cells. Thus, the exact role of NRF2 in T cells and T cell subsets in autoimmunity is not clear.

## Redox Metabolism in SLE T Cells

ROS production and cellular metabolism are intimately linked. More interestingly NRF2 is also involved in the regulation of different metabolic pathways. For immune cells, NRF2 increased glucose uptake and mitochondrial function, while reducing ROS in natural killer (NK) T cells and myeloid derived suppressor cells (MDSCs) (25, 51). Immune cells undergo metabolic reprogramming in autoimmune and autoinflammatory diseases. This is also the case in SLE, where T cells are hyper-oxidative (52). T cells from SLE patients differ from activated T cells from healthy persons (53, 54). Activated T cells mostly use glycolysis and pentose phosphate pathway to produce ATP and metabolic intermediates. SLE T cells show a chronically activated phenotype with increased TCA (trichloroacetic acid) cycle activity, they furthermore depend on OXPHOS to fulfill their energetic demands (55). It is suggested that SLE T cells therefore have lower antioxidant capacity with lower NADPH and glutathione levels (1, 56). This is further indicated by the fact that SLE T cells display high oxidative stress (57, 58). Although SLE T cells have an enlarged mitochondrial mass, the ATP generation from OXPHOS is insufficient compared to healthy person and a marked leakage of ROS is present in SLE T cells (58). ROS increase mammalian Target of Rapamycin (mTOR) activity, which is increased in CD4^+^ T cells in SLE patients (59). So reduced GSH levels induce a redox-dependent mTOR activation. CD4^+^ T cells from lupus patients show enhanced levels of mTOR activation which has been mechanistically linked with the disease process (60). As a consequence, therapeutic interventions with mTOR inhibitors in SLE patients normalized T cell activation and improved their clinical disease activity (61, 62).

There seem to be T cell subtype specific regulations that also need to be considered. Recently, Cailelli et al. identified CD4^+^ T cells that are induced by oxidized mitochondrial DNA-activated pDCs in SLE patients and accumulate ROS, secrete succinate and produce IL-10 and IFN-γ. By doing so, they provide superb B cell help (63).

This suggests that ROS accumulation plays a pathogenic role in SLE T cells. As a consequence, enhancing NRF2 activity and thereby reducing intracellular redox metabolism could be beneficial.

## Discussion

**Figure 1:**
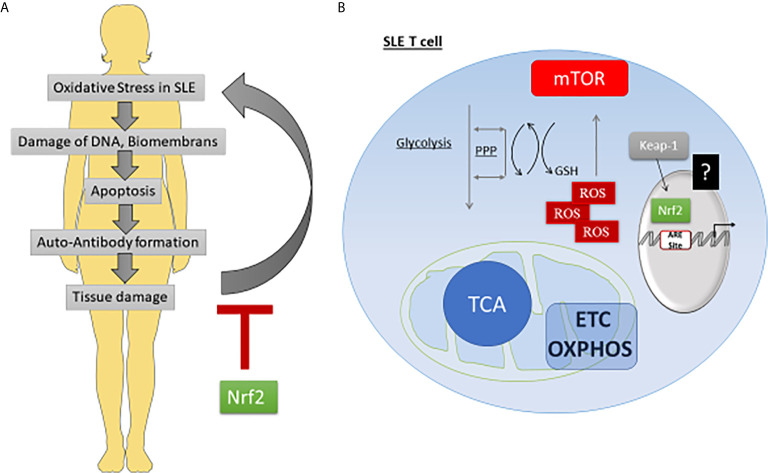
While systemic NRF2 activation can ameliorate pathogenesis, the exact role in T cells is not clear. **(A)** Oxidative stress is involved in SLE pathogenesis. Due to increased free radicals or a weak antioxidant system, SLE patients reveal high levels of oxidative stress which leads to cell damage and apoptosis. Subsequent release of autoantigens can enhance autoantibody formation and contribute to tissue damage. Tissue damage again can enhance oxidative stress. Several lines of evidence indicate that activated NRF2 can break the vicious circle by inducing anti-oxidative responses. **(B)** SLE T cell are hyper-oxidative. SLE T cells prefer the use of OXPHOS, which reduces NADPH and GSH pools, that are normally filled up during glycolysis and pentose phosphate pathway (PPP). In addition, they reveal a marked leakage of ROS during OXPHOS. High ROS levels induce mTOR activation. It is not clear, if NRF2 is not sufficiently activated or somehow fails to counteract high ROS levels in SLE T cells.

Without a doubt redox metabolism critically regulates function, proliferation, and apoptosis of T cells. Furthermore, oxidative stress is a hallmark of SLE pathogenesis and T cells from SLE patients reveal metabolic aberrations that also involve higher ROS production. Therefore NRF2, as main transcriptional regulator of the anti-oxidative stress response and beyond this as regulator of metabolic and anti-inflammatory actions in immune cells, is most likely a central player in abnormal SLE T cell function. In line, 509 unique patent applications that define NRF2 pathway as molecular target and focus on medical conditions such as autoimmunity, liver, kidney, lung and neurodegenerative diseases have been filed since 2017 (64). Furthermore dimethyl fumarate (DMF), which activates Nrf2, is already used clinically to treat inflammatory diseases. DMF was approved for the treatment of patients with moderate and severe psoriasis as well as patients with relapsing remitting multiple sclerosis (65). In addition, there are clinical trials ongoing with DMF for treatment of inflammatory conditions, among others cutaneous lupus erythematosus (66). On the other hand, medication currently in use to treat patients with SLE clearly seems to affect Nrf2 signaling. In particular corticosteroids, which are first line treatment drugs downregulate Nrf2 transcriptional activation by direct and indirect means (67–69). Moreover, Cyclophosphamide, which is used in severe cases (lupus nephritis and CNS lupus) has severe side effects like hepatotoxicity and myelosuppression, which can be ameliorated by activation of NRF2 (70, 71). The same is true for methotrexate, which is used in lupus arthritis and downregulates the antoxidative Nrf2 response in the liver (72). On the other hand, mycophenolate mofetile is increasingly used in SLE and preserves the Nrf2 system in the liver as well as in the kidney, which might contribute to the broad therapeutic tolerability of the drug (73, 74). Finally, hydroxychloroquine also preserves the Nrf2 antioxidative system (75). Nevertheless, the described effects have mainly been shown in other systems than T cells and the immune system (liver, kidney and cancer models) and therefore we can only speculate on their effects on immune functions. Although it is well established that NRF2 has anti-inflammatory effects, the cellular mechanism and cell-specific actions are still not fully elucidated. Several controversies exist regarding the ambivalent role of ROS production in autoimmune diseases and the pathogenic effects of ROS are supposed to be dependent on threshold, location, and time. Furthermore, the cellular compartment seems to be of importance. While a global NRF2 activation is beneficial in autoimmune diseases, it is not clear if NRF2 has anti-inflammatory roles specifically in T cells (**Figure 1**). Future experiments might focus on T cells and T cell subtype specific roles of NRF2. Noticeable, SLE T cells display an altered redox metabolism. N-Acetylcysteine reversed glutathione depletion and thereby blocked mTOR activation in T cells and improved disease activity in SLE patients (76). One main problem of SLE patients is their susceptibility to infections. The use of glucocorticoids as immunosuppressive therapy increases this risk. Instead of completely blocking and suppressing the immune system a tight modulation of inflammation while preserving the cells’ overall functionality would be much more desirable to treat autoimmune diseases. Therefore, a therapeutic control of NRF2 activity might be a starting point to influence Redox metabolism in SLE T cells, but in-depth analysis of pathways and possible side-effects is absolutely necessary.

## Author Contributions

KO and KT agree to be accountable for the content of the work. All authors contributed to the article and approved the submitted version.

## Funding

This review was partially supported by the Deutsche Forschungsgemeinschaft (DFG OH 252/2‐1).

## Conflict of Interest

The authors declare that the research was conducted in the absence of any commercial or financial relationships that could be construed as a potential conflict of interest.

## Abbreviations

DAMPs, danger-associated molecular patterns; ETC, electron transport chain; GCLC, Glutamate-Cysteine Ligase Catalytic Subunit; GPX, gluthathione peroxidase; GSH, gluthathione; GvHD, graft-vs-host disease; Keap1, Kelch ECH associating protein 1; MDSC, myeloid derived suppressor cells; mTOR, Mammalian Target of Rapamycin; NCF, Neutrophil Cytosolic Factor; NK, natural killer; NOX, NADPH oxidase; NQO1, NAD(P)H: quinone oxidoreductase 1; Nrf2, Nuclear factor erythroid 2-related factor 2; OXPHOS, oxidative phosphorylation; PBMCs, peripheral blood mononuclear cells; ROS, rective oxygen species; SLE, systemic lupus erythematosus; SOD, superoxid dismutase; TCA, trichloroacetic acid.
